# Bruegel’s *Landscape with the Fall of Icarus*

**DOI:** 10.14797/mdcvj.1377

**Published:** 2024-05-16

**Authors:** Justin C. Cordova, James B. Young

**Affiliations:** 1 Department of Anesthesiology, Walter Reed National Military Medical Center, Bethesda, Maryland, US; 2 Emeritus Executive Director of Academic Affairs, Cleveland Clinic and Professor Emeritus of Medicine, Cleveland Clinic Lerner College of Medicine of Case Western Reserve University, Cleveland, Ohio, US; Section Editor, Poet’s Pen, *Methodist DeBakey Cardiovascular Journal*

**Keywords:** pain, grief, tragedy, ekphrastic poetry

## Abstract

Ovid’s *Metamorphoses* tells the story of Icarus – his tragic flight with man-made wings, the melting of the wax that bound them, and the ensuing fall to his death. This moment has been immortalized across the arts and through several mediums, but none are more notable than Bruegel’s *Landscape with the Fall of Icarus*. Described as a “painter for poets,” Bruegel’s work served as inspiration for several writers, with this piece in particular providing the basis for ekphrastic poems by W.H. Auden and William Carlos Williams. Though each of these works has a different focus, the unifying theme is that human tragedy is too often placed on the periphery of notice. They are effective reminders to physicians and other healthcare providers about the human aspect of suffering and pain in medicine.

## Musée des Beaux Arts

In Breughel’s *Icarus*, for instance: how everything turns away

Quite leisurely from the disaster…

Something amazing, a boy falling out of the sky….

W. H. Auden

From *Collected Poems of W.H. Auden, ed. Edward Mendelson* (New York: Viking Press: 1991)^1^

## Landscape with the Fall of Icarus

…when Icarus fell

it was spring…

there was

a splash quite unnoticed

this was

Icarus drowning.

William Carlos Williams

From *The Collected Poems of William Carlos Williams: Volume II, 1939-1962, ed MacGowan C*. (New York: New Directions Publishing Corp: 1962)^2^

## Commentary

The tale of Icarus is found in the eighth book of Ovid’s *Metamorphoses*, just after the story of the Minotaur and before the Calydonian boar hunt. He and his father, Daedalus, were trapped on the island of Crete and turned to the skies as a means to escape their imprisonment. Daedalus fashioned wings for himself and his son using feathers, attaching them with beeswax and thread. He instructed his son not to fly too close to the sea lest moisture weigh down the feathers, nor too near the sun lest his wings be scorched by its heat. Though he initially chose a safe passage, Icarus had “fallen in love with the sky, and soared up higher and higher”^3^ until the wax holding his wings melted in the sun’s heat. Icarus fell to his death in the sea, near an island where his fall may have been noticed by an angler catching fish, a plowman resting on his plow, or a shepherd leaning on his crook. Daedalus was devastated; this “unhappy father, no longer a father,”^3^ buried his son’s body in a tomb on the island, naming it Icaria in his honor, which can be found in the Aegean Sea as part of modern-day Greece.

The fall of Icarus has been portrayed by a number of artists, including Peter Paul Rubens and Anthony van Dyck. This oil on canvas, titled *Landscape with the Fall of Icarus*, has been attributed to Pieter Bruegel the Elder (1525-1569), a Flemish Renaissance painter known for his landscapes and narrative scenes. Many of his pieces were reimaginings of biblical or mythological events, some of which featured similar portrayals of personal hardship alongside a universal tendency to continue everyday life.^4^ This painting shows Icarus as nothing more than a pair of legs, intentionally placed away from the focal point of the canvas, which more prominently features a stunning landscape and the shepherd, plowman, and fisher that Ovid describes. Bruegel (also spelled Breughel or Brueghel) is thought to have created his version in 1555, though the original is neither signed nor dated and its authorship continues to be debated.^5^ The piece was discovered in 1912 and bought by the Royal Museums of Fine Arts of Belgium in Brussels, where it was seen by W.H. Auden in 1938 and by William Carlos Williams in the 1950s and where it remains today.

**Figure:**
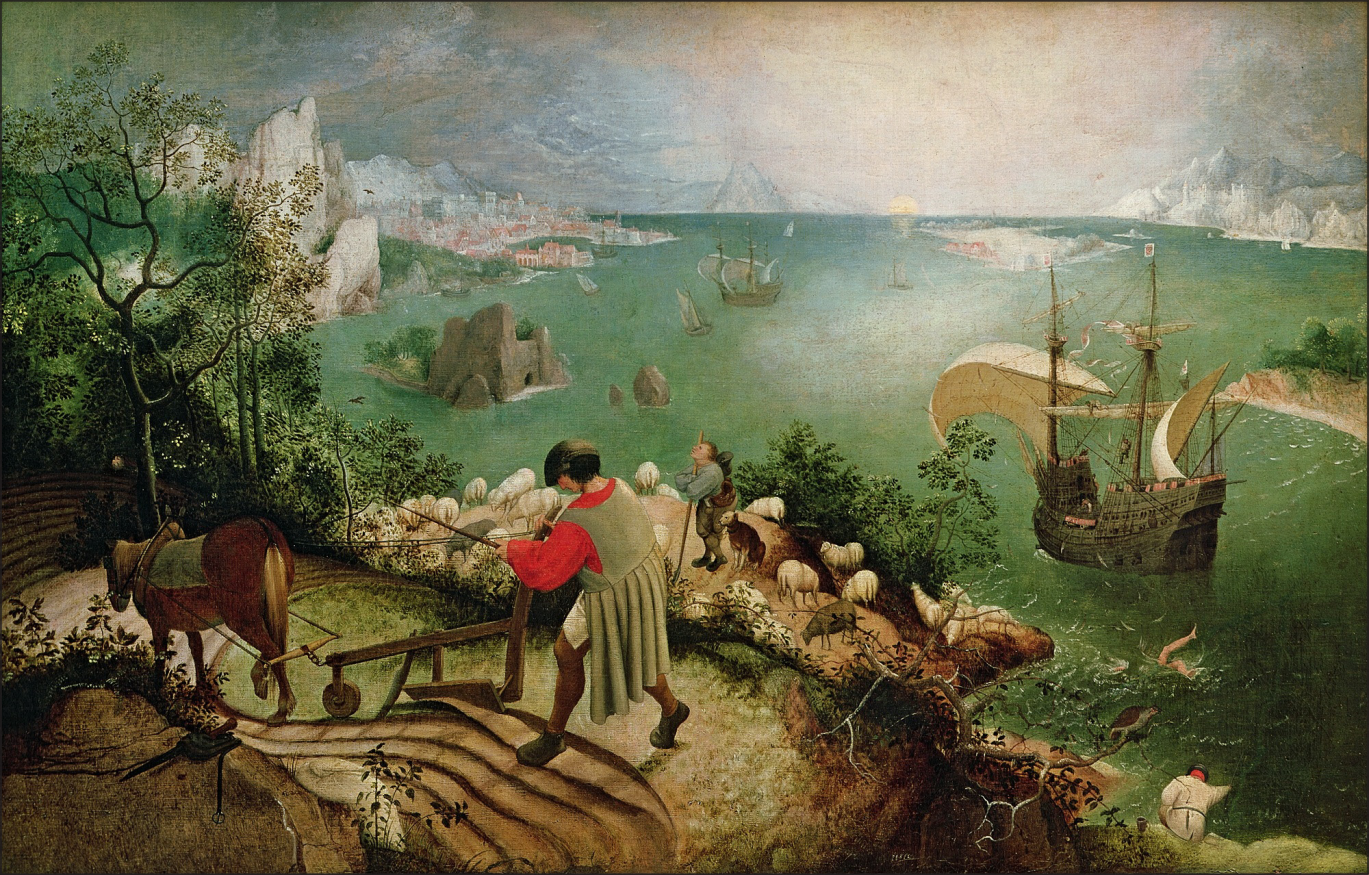
*Landscape with the Fall of Icarus*, attributed to Pieter Bruegel the Elder (c. 1555). Source: Bridgeman Art Library: Object 3675, Public Domain, https://commons.wikimedia.org/w/index.php?curid=11974918.

W.H. Auden (1908-1973) was an important poet of his time and continues to impact readers more than 50 years after his death. Born in England, Auden traveled widely before settling in the United States. He received the Pulitzer Prize for *Age of Anxiety* in 1948 and published dozens of volumes of poetry, prose, and essays throughout his much decorated career.^6^ In “Musée des Beaux Arts,” Auden recounts the visit he made to the museum in Brussels, with the latter half of the poem describing his reaction to *Landscape with the Fall of Icarus*. The first stanza may describe other works by Bruegel, which are displayed in an adjacent alcove dedicated to the artist, but Auden doesn’t identify them by name. What struck the poet so clearly is how the world continues to function in spite of misery and anguish, how “everything turns away/Quite leisurely from the disaster.”^1^ It seems as though Auden has distilled the message that Bruegel was hoping to portray, highlighting the nonchalance with which the world greets tragedy, perhaps in hope that his words could lead it to do otherwise.

William Carlos Williams (1883-1963) had a similar reaction when he saw the painting in the 1950s, though one can only speculate as to whether he had read or been influenced by Auden’s piece from nearly two decades before. Williams was the quintessential physician-writer and one of few such physicians to gain critical acclaim for his literary work. Born in Rutherford, NJ, Williams returned there to practice after completing medical school at the University of Pennsylvania School of Medicine, along with further training in New York City and Leipzig, Germany. Though he began writing poetry at an early age, Williams was halfway through his medical training before he started writing in earnest. He came to discover that writing was his true calling, while medicine was the vehicle by which he could sustain and support his literary passions.^7^ In “Landscape with the Fall of Icarus,” Williams focuses on the glory and indifference of nature, reflecting on the “pageantry of the year” that was on display while a barely perceptible splash signaled the end of Icarus’ tragic descent. The poem is typical of the sparse and experimental writing style for which he would be hailed the “godfather of avant-garde poetry.” It was part of his collection, *Pictures from Brueghel and Other Poems* (1962), for which he posthumously received the Pulitzer Prize in 1963.

These three works, two ekphrastic poems and one masterful painting, are poignant reminders of human suffering, particularly that which is inherent to the practice of medicine. There is a personal aspect of pain and grief that should not be forgotten, downplayed, or discounted. In each piece, individual calamity is not front and center; lives go on despite the anguish contained in the scene. Medical providers are unavoidably close to mortality on a daily basis; they do not have the luxury of looking away from center stage but are instead at risk of being inured to its consequences. Works of art and literature are a subtle reminder of the human aspect of this grief, which Bruegel, Auden, and Williams represent so well. Physicians and other practitioners of medicine must find a way to share this grief with patients and their families to process poor outcomes on a personal level and to prepare for their next encounter with those who are suffering.

## Disclaimer

The views expressed are solely those of the authors and do not reflect the official policy or position of the US Army, US Navy, US Air Force, the Department of Defense, the US Government, or Houston Methodist Hospital.
